# Mobile Genetic Elements as Key Drivers of Bacterial Evolution and Adaptation in Agroecosystems

**DOI:** 10.1007/s00248-026-02803-5

**Published:** 2026-06-12

**Authors:** Manuela Costanzo, Luciana Di Gregorio, Silvia Tabacchioni, Annamaria Bevivino, Andrea Visca

**Affiliations:** Department for Sustainability, ENEA, Rome, Italy

**Keywords:** Horizontal gene transfer, Genome plasticity, Mobile genetic elements, Agroecosystems, Bacterial evolution

## Abstract

Mobile genetic elements (MGEs), including plasmids, transposons, integrative and conjugative elements, and phage-derived sequences, are central drivers of bacterial evolution in agroecosystems. By enabling horizontal gene transfer, MGEs allow soil- and plant-associated bacteria to rapidly acquire complex functional traits, facilitating adaptation to fluctuating environmental conditions and different agricultural management practices. In agricultural soils, MGEs underpin key microbial functions such as nutrient acquisition and cycling, stress tolerance, rhizosphere competence, and interactions with plant hosts, thereby influencing soil fertility and crop performance. Selective pressures in agroecosystems extend beyond antimicrobial exposure and include fertilizers, pesticides, plant defense compounds, recurrent biotic and abiotic stress, as well as high-yielding crop varieties. These pressures generate co-selection dynamics that shape mobilome composition and activity, linking traits such as resistance, pathogenicity, and biocontrol to broader ecological functions relevant to plant health. Rather than acting as exceptional genetic entities, MGEs form a dynamic and environmentally responsive genetic network that enables rapid ecological tuning while preserving core genome stability. Comparative genomics has revealed that major lifestyle transitions in agroecosystem-associated bacteria, from free-living to commensal, mutualistic, or pathogenic states, are frequently mediated by the gain and loss of genomic islands and other MGEs. This review synthesizes the latest research on the ecological functions and evolutionary dynamics of MGEs in agroecosystems and explores how mobilome-informed approaches can support microbial-based strategies for sustainable agriculture.

## Introduction

### What are Mobile Genetic Elements

Mobile genetic elements (MGEs) are discrete DNA entities capable of moving within a genome or transferring between bacterial cells, thereby mediating horizontal gene flow across populations and environments [1]. They encompass a diverse set of genetic platforms, including plasmids, transposons, insertion sequences, integrons, bacteriophages, integrative and conjugative or mobilizable elements (ICEs and IMEs), as well as more recently recognized entities such as phage-inducible chromosomal islands (PICIs), satellite phages (cf-PICIs), and composite defense-associated genomic islands. Despite their structural and mechanistic diversity, all MGEs share the capacity to decouple gene inheritance from vertical descent, allowing genetic traits to spread independently of clonal reproduction [2]. Plasmids are extrachromosomal replicons that vary widely in size, copy number, and host range, and may be conjugative, mobilizable, or non-transmissible. Even when not self-transferable, plasmids frequently persist through stable inheritance or hitchhiking with other mobile elements, acting as long-term reservoirs of adaptive genes [3]. Transposons and insertion sequences mediate genome plasticity by relocating DNA segments within and between replicons, often mobilizing accessory genes or reshaping regulatory architectures [4]. Integrons function as gene capture and expression systems, assembling modular gene cassettes that can be rapidly reorganized in response to selective pressure, particularly when embedded within plasmids or transposons [5, 6]. Bacteriophages represent a major but often underestimated component of the mobilome. Through lysogeny, prophages introduce auxiliary genes that can modulate host fitness, stress tolerance, or ecological interactions. Beyond classical prophages, PICIs and cf-PICIs act as phage satellites that exploit helper phages for mobilization while redirecting phage replication and packaging. These elements are increasingly recognized as highly efficient vectors for the dissemination of virulence factors, metabolic traits, and defense systems [7]. ICEs and IMEs occupy an intermediate position between plasmids and chromosomal islands, integrating into specific chromosomal sites while retaining the ability to excise and transfer via conjugation [8]. Together, these elements form the mobilome, a dynamic and interconnected genetic layer that overlays the bacterial chromosome and links genomes into networks of horizontal exchange. Rather than isolated entities, MGEs frequently interact, recombine, or co-localize, giving rise to composite regions that combine mobility, defense, and fitness-related functions [9].

### Why MGEs are Central to Bacterial Evolution

MGEs profoundly alter the *tempo* and mode of bacterial evolution by enabling the acquisition of complex traits in single evolutionary steps [10]. In contrast to point mutations, which generate incremental variation, MGEs can mobilize entire operons, gene clusters, or regulatory modules, immediately conferring new phenotypes [11]. This capacity allows bacteria to rapidly respond to selective pressures such as nutrient limitation, chemical stress, competition, or host-associated challenges [12]. Beyond gene acquisition, MGEs actively restructure genomes. Transposition, site-specific recombination, and prophage integration generate insertions, deletions, inversions, and hybrid genetic architectures that rewire regulatory networks and expand phenotypic diversity [13, 14]. These structural changes are not random but often occur at permissive genomic loci, leading to the stabilization of horizontally acquired traits. Over time, some MGEs become domesticated, losing mobility while retaining functions beneficial to the host, such as toxin–antitoxin systems, stress-response modules, or phage resistance mechanisms [1]. Evolutionary outcomes are shaped by the balance between the costs imposed by MGEs and the benefits they provide [10]. While MGEs may reduce host fitness through metabolic burden or genomic instability, they also supply adaptive traits that enhance survival under fluctuating environmental conditions. This tension has driven the emergence of defense islands, genomic regions enriched in restriction–modification systems, abortive infection modules, toxin–antitoxin systems, and related functions [15]. These islands are themselves frequently mobile or assembled through MGE-mediated processes, highlighting that defense against genetic parasites is deeply embedded within the same evolutionary framework that promotes genetic exchange. At the population and community level, MGE-mediated gene flow blurs species boundaries and fuels pangenome expansion [16]. Core genomes provide lineage identity and evolutionary stability, whereas accessory and cloud genes, largely mobilome-associated, enable rapid ecological specialization [17]. Through repeated cycles of acquisition, selection, and loss, MGEs contribute to lineage diversification, ecotype formation and, in some cases, the emergence of novel pathogenic or symbiotic lifestyles [18].

### Importance of Studying MGEs in Agroecosystems

Agroecosystems represent some of the most dynamic and intensively managed microbial habitats on Earth, characterized by recurrent disturbances, strong anthropogenic selection pressures, and dense microbial communities [19]. Soil, rhizosphere, phyllosphere, and endosphere microbiomes are continuously exposed to fertilizers, pesticides, heavy metals, antibiotics and fluctuating nutrient inputs, creating ideal conditions for MGE-mediated adaptation [20–22]. In these environments, MGEs play a central role in shaping microbial functionality. Plasmids and ICEs disseminate genes involved in nutrient acquisition, plant polymer degradation, phytohormone modulation, and stress tolerance [23, 24]. Transposons and integrons contribute to the assembly of adaptive gene combinations, often coupling resistance determinants with metabolic or colonization traits [25]. Phages, PICIs and related satellite elements modulate population dynamics, influence competition and act as vectors for both adaptive and defensive functions [26, 27]. Importantly, agroecosystems function as critical interconnected interfaces between environmental (soil/water), animal and human microbiomes [28]. MGEs circulating in agricultural settings can link soil and plant-associated bacteria with livestock and clinical reservoirs, enabling resistance and virulence genes to cross ecological boundaries through horizontal gene transfer and ecological connectivity between microbial communities [29]. At the same time, beneficial MGEs underpin traits relevant to sustainable agriculture, such as biological control, plant growth promotion and resilience of microbial inoculants [30]. Understanding MGEs in agroecosystems therefore has dual relevance: mitigating risks associated with undesirable gene flow and harnessing natural genetic mobility to design robust, adaptable microbial solutions. Viewing agricultural microbiomes through a mobilome-centered lens shifts the focus from static community composition to dynamic genetic potential.

### Objective of this Review

**Fig. 1:**
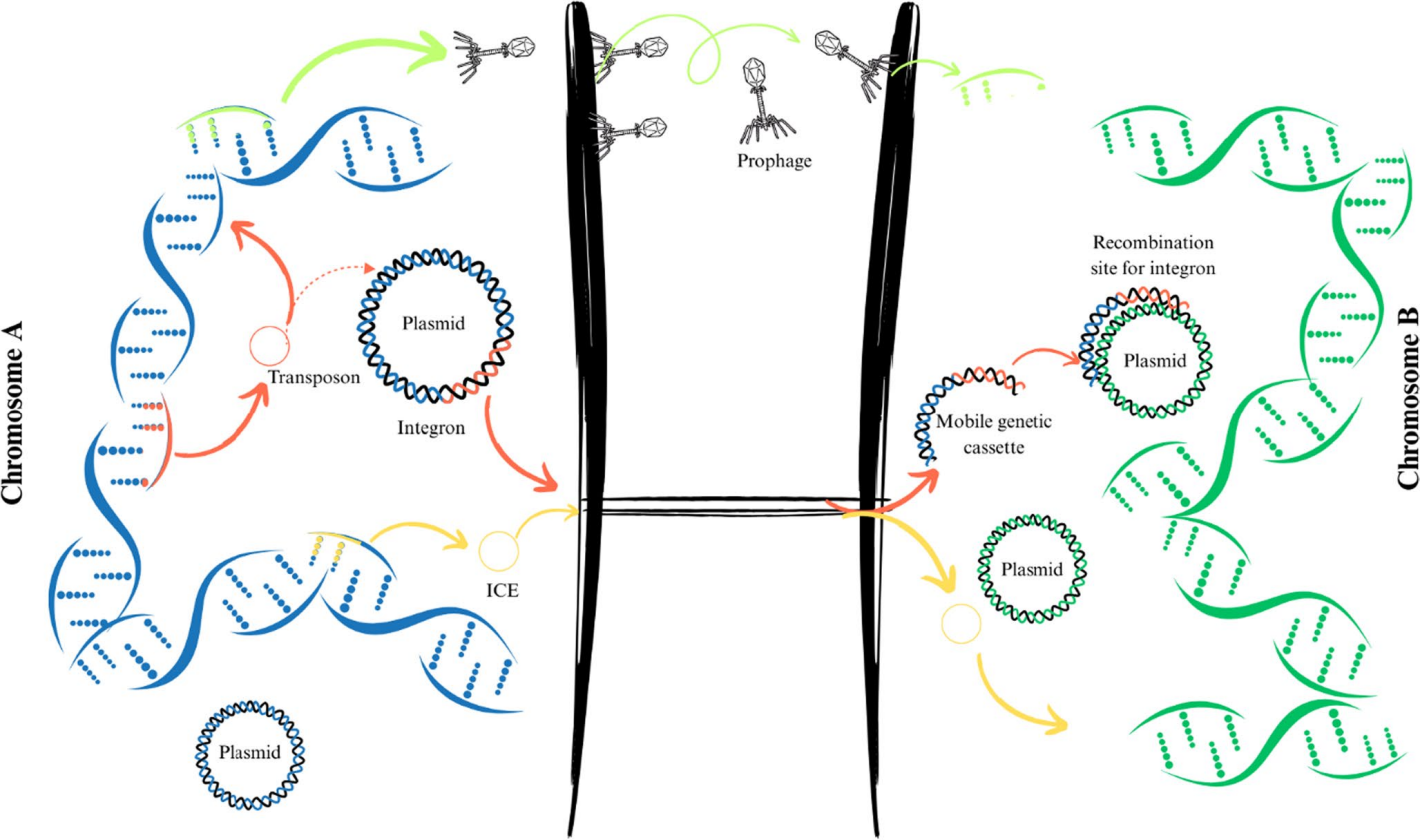
Mobile genetic elements as drivers of horizontal gene transfer and genome modularity in bacteria. The schematic illustrates the major classes of mobile genetic elements and their interactions across bacterial chromosomes. Chromosomal DNA (Chromosome A and B) coexists with plasmids, integrons, transposons, integrative and conjugative elements (ICEs), and bacteriophages (prophages), which collectively mediate horizontal gene transfer (HGT). Transposons and integrons mobilize gene cassettes between plasmids and chromosomes, while ICEs enable chromosomal excision, transfer, and reintegration across cells. Plasmids act as central hubs for the acquisition, recombination, and dissemination of adaptive traits, including resistance and metabolic functions. Phage-mediated transduction further contributes to intercellular gene flow. Together, these processes create a dynamic genomic network in which genes continuously transition between chromosomal and extrachromosomal compartments, underpinning genome plasticity, rapid adaptation, and the assembly of accessory and cloud genomes across bacterial populations

This review aims to reposition mobile genetic elements as key drivers of bacterial evolution in agroecosystems, rather than merely as vectors of antibiotic resistance. Integrating genomic and ecological perspectives, we examine how diverse classes of MGEs, including plasmids, transposons, integrons, integrative and conjugative elements, bacteriophages, PICIs, cf-PICIs, as well as defense-associated genomic islands, collectively shape bacterial adaptation in agricultural environments. Specifically, we first introduce the main functional categories of MGEs and their mechanisms of mobility, followed by dedicated sections that illustrate their roles through representative case studies in agroecosystems, highlighting both canonical and emerging systems. Within this framework, antibiotic resistance is interpreted as one outcome of a broader mobilome-driven evolutionary logic. Mobile genetic elements play a central role in the environmental dissemination of antibiotic resistance genes across agroecosystems [31, 32]. In livestock-associated settings, manure, slurry, and wastewater act as major reservoirs where plasmids, integrative elements, and phage-related particles mediate high-frequency horizontal gene transfer [7, 33, 34]. Upon field application, these matrices introduce MGEs into soil microbiomes, where they can persist, recombine, and spread across taxonomically diverse bacterial populations [35, 36]. This establishes a continuum between agricultural, environmental, and clinical resistomes, reinforcing the role of agroecosystems as critical nodes in the global transmission network of antimicrobial resistance. Our focus is on how MGEs drive the acquisition, stabilization, and dissemination of traits relevant to survival, competitiveness, and ecological performance in agroecosystems. Particular attention is given to the relationship between genetic mobility and defense systems, stress tolerance, and functional specialization, highlighting MGEs as both evolutionary catalysts and ecological regulators (Fig. 1).

## MGEs in Agroecosystems: Insights From Recent Case Studies

### Plasmids as Key Evolutionary Drivers of Bacterial Adaptation in Agroecosystems

Agricultural soils and plant-associated habitats are heterogeneous and frequently disturbed systems, where bacteria experience fluctuating selective pressures driven by farming practices such as manure application, pesticide use, chemical fertilization and metal inputs. In these environments, plasmids contribute to bacterial evolution by enabling rapid genetic exchange and maintaining adaptive potential under changing conditions.

A key feature of many soil plasmids is their ability to disseminate genes across phylogenetically diverse hosts. Broad-host-range (BHR) plasmids are particularly relevant in agroecosystems [37, 38], as their transfer range often exceeds their replication range, allowing them to act as genetic shuttles across taxonomic boundaries. This explains the widespread distribution of identical resistance genes in both Gram-positive and Gram-negative bacteria. Their success relies on host-independent replication and tight regulation of gene expression, which minimizes fitness costs while preserving transfer capacity [39]. Repression of conjugation genes, for example, balances dissemination with host fitness and may reduce susceptibility to phage predation [40].

Comparative genomics shows that many soil plasmids share conserved backbones for replication, maintenance, and transfer, combined with variable accessory regions encoding adaptive traits [41, 42]. IncP-1 plasmids exemplify this structure and are widespread in soils, manure, and rhizospheres [43]. Their conserved backbone and flexible gene cargo support a model in which plasmids act as stable platforms that intermittently acquire adaptive functions via recombination and transposition [36]. However, their persistence is strain-dependent, indicating that host permissiveness to plasmid carriage is itself under selection [44].

Agroecosystems also harbor diverse environmental plasmids with less obvious functions. PromA-like plasmids, frequently found in rhizosphere bacteria [45–47], are highly conserved and efficient at mobilizing other plasmids [46], suggesting a role in maintaining access to the horizontal gene pool. In contrast, IncP-9 plasmids are often associated with polluted soils and encode catabolic pathways for aromatic compounds [48], highlighting how plasmids can facilitate adaptation to anthropogenic inputs.

Under strong selection, such as antibiotic or metal exposure, plasmids play a major role in maintaining adaptive traits. Manure-amended soils often select for plasmids carrying multiple resistance genes organized in mosaic regions [49, 50], promoting co-selection. Similarly, metal-contaminated soils enrich plasmids encoding mercury or multi-metal resistance, where spatial heterogeneity supports coexistence of plasmid-bearing and plasmid-free populations [51].

**Fig. 2:**
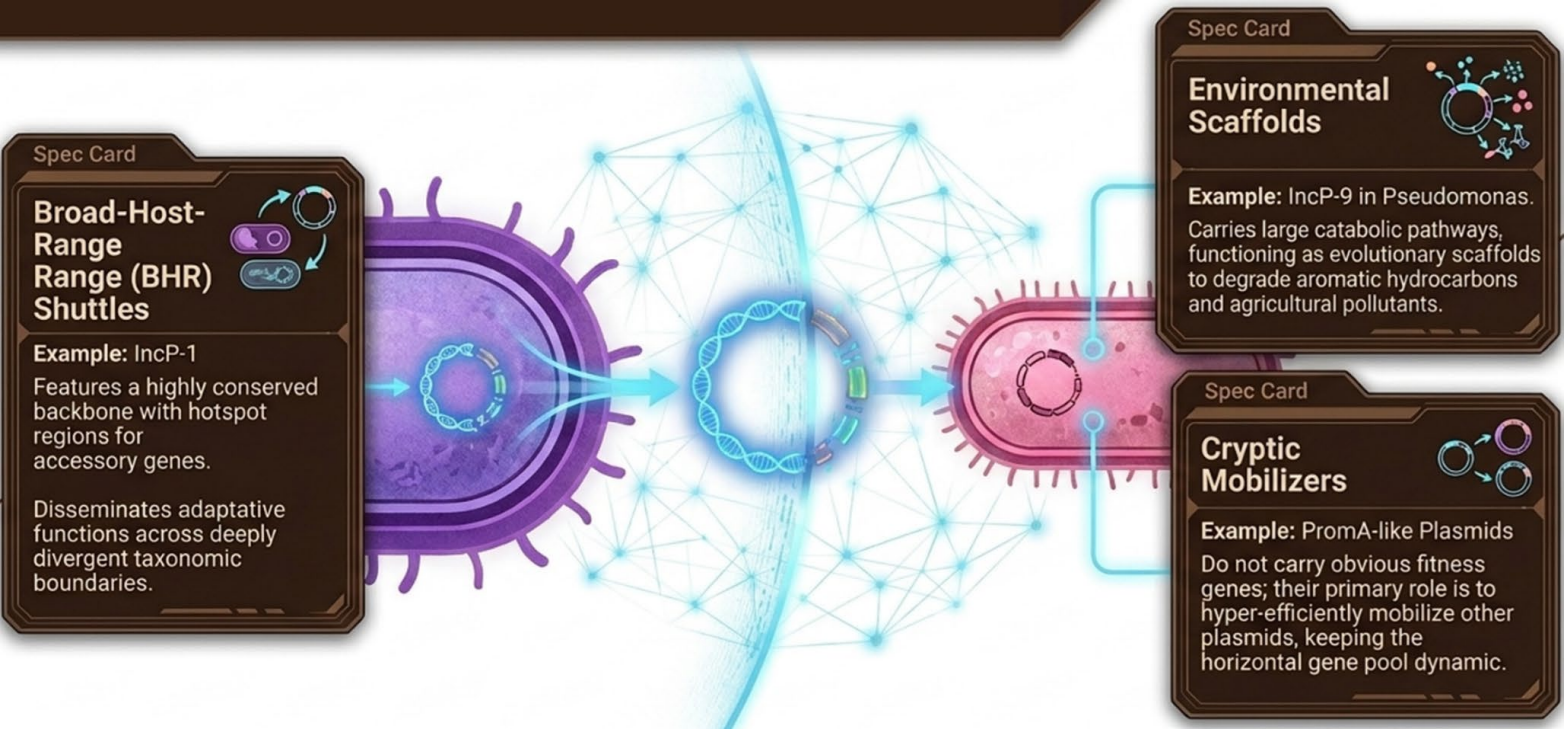
Conceptual model of plasmid-driven adaptation in agroecosystems. Environmental stressors (pesticides, metals, crop management) select for plasmid-carried traits, including antibiotic resistance, catabolic pathways, and regulatory functions. Broad-host-range plasmids (IncP-1, IncP-9) facilitate horizontal gene transfer across diverse bacteria, while cryptic plasmids can influence host interactions. This plasmid-mediated gene flow creates heterogeneous, robust populations capable of rapid evolution under fluctuating conditions

Although plasmids can impose fitness costs [52, 53], they persist because they increase population-level adaptability in fluctuating environments [54–56]. Rather than benefiting every cell, plasmids maintain standing genetic variation, allowing rapid responses to environmental change and supporting long-term ecological resilience (Fig. 2).

### Integrative and Conjugative Elements in Plant-associated Bacteria: Evolutionary Implications in Agroecosystems

Integrative and conjugative elements (ICEs) are widespread in plant-associated bacteria and represent major contributors to genome evolution in agroecosystems [57]. Their dual ability to integrate into chromosomes and transfer between cells enables long-term persistence while maintaining horizontal mobility.

In plant pathogens, ICEs contribute to adaptation by carrying genes involved in virulence, stress response, and resistance. In *Agrobacterium tumefaciens*, ICEs are unevenly distributed and often integrate into tRNA genes, reflecting horizontal acquisition followed by genomic stabilization [58, 59]. Their cargo includes functions related to plant interaction and environmental survival [58]. Similarly, ICEs in *Dickeya* and *Pectobacterium* species display conserved core modules but variable accessory regions encoding secretion systems, resistance traits, and virulence factors [51]. High ICE diversity is observed in *Pseudomonas syringae*, where multiple elements can coexist within the same genome [51]. These ICEs frequently encode resistance to metals such as copper and arsenic [24, 60], as well as Type III secretion system effectors [61], reflecting adaptation to agrochemical exposure and host interactions. Comparable patterns occur in *Ralstonia solanacearum*, *Xanthomonas campestris*, and *Xylella fastidiosa*, where ICEs contribute to stress tolerance and pathogenicity while maintaining conserved backbone structures [51, 62, 63].

Overall, ICEs play a central role in bacterial evolution in agroecosystems by stabilizing adaptive traits within genomes while enabling their horizontal spread under favorable conditions. Through the acquisition and redistribution of genes involved in virulence, resistance, and environmental fitness, ICEs facilitate rapid ecological adaptation and contribute to the evolutionary plasticity of plant-associated bacteria.

**Fig. 3:**
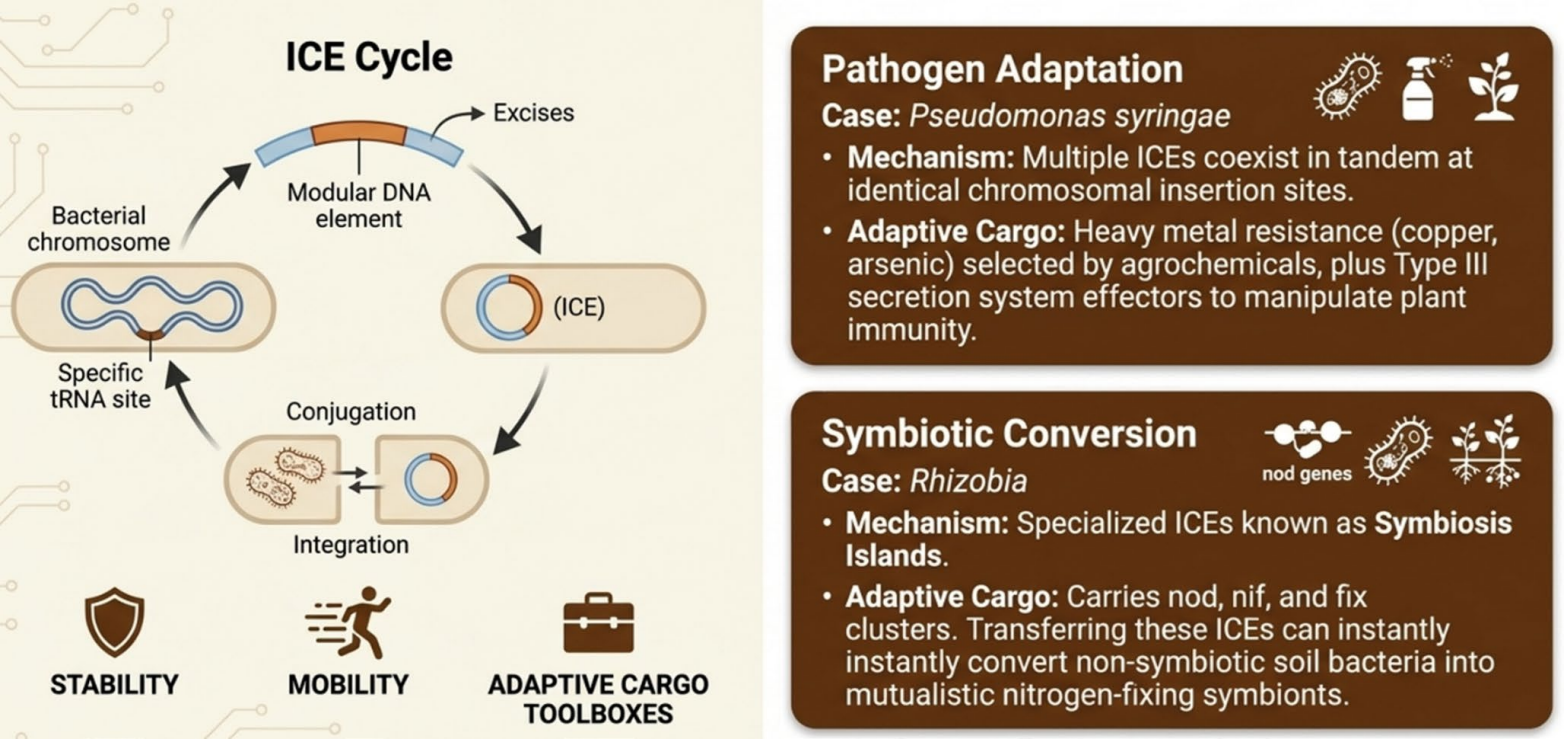
ICE-mediated evolution in agricultural bacteria. ICEs facilitate horizontal transfer of adaptive traits (resistance, virulence, metabolism) among bacteria. Their ability to integrate into chromosomes ensures persistence, while conjugation enables dissemination across populations. In agroecosystems, ICEs respond to selective pressures (chemical inputs, host interactions) by reshaping the genetic landscape of soil microbiomes and pathogens, driving rapid adaptation and ecological change

While ICEs have been extensively characterized in plant pathogens, they are equally widespread and functionally important in non-pathogenic, plant-associated bacteria, including endophytes, rhizosphere colonizers, and symbiotic nitrogen fixers. In these systems, ICEs contribute not to virulence but to ecological competence, metabolic versatility, and host interaction capabilities. In rhizobia, for instance, large symbiosis islands, often considered specialized ICEs, carry genes required for nodulation and nitrogen fixation, including *nod*, *nif*, and *fix* clusters, enabling the establishment of mutualistic interactions with leguminous plants [64, 65]. These elements can be horizontally transferred between strains, effectively converting non-symbiotic soil bacteria into symbionts and reshaping the functional composition of rhizosphere communities. Beyond classical symbiosis, ICEs in plant-beneficial bacteria frequently encode traits that enhance rhizosphere fitness, such as secretion systems, siderophore production, stress response pathways, and metabolic functions involved in root exudate utilization [66]. In endophytic and rhizospheric populations, ICE-mediated gene transfer facilitates rapid adaptation to fluctuating environmental conditions and host-derived selective pressures, including plant immune responses and nutrient availability [67, 68]. This is particularly relevant in agroecosystems, where recurrent disturbances such as fertilization, irrigation, and crop rotation impose strong selection on microbial communities [69]. Importantly, ICE dynamics in these bacteria often interact with other components of the mobilome, including plasmids and genomic islands, contributing to a highly interconnected network of gene exchange. Comparative genomic studies have revealed that closely related rhizobial strains can differ markedly in plasmid and ICE content, suggesting ongoing acquisition, loss, and recombination of mobile elements as key drivers of functional diversification [70]. This genomic plasticity underpins the emergence of strains with distinct ecological strategies, ranging from highly specialized symbionts to more versatile, free-living soil bacteria. Taken together, these observations highlight that ICEs are not merely vectors of pathogenicity, but central components of adaptive evolution in beneficial plant-associated bacteria. Expanding the focus beyond pathogenic systems is therefore essential to fully understand how MGEs shape microbial functions that are directly relevant to soil fertility, plant productivity, and the sustainability of agricultural systems (Fig. 3).

### Transposons and Insertion Sequences As Drivers of Genome Plasticity in Agroecosystem-associated Bacteria

Transposons (Tns) and insertion sequences (ISs) are major contributors to genome plasticity in plant-associated bacteria. By mediating gene disruption, recombination, and horizontal transfer, they shape bacterial evolution under agricultural selective pressures [71]. Their distribution varies across taxa. While IS5 elements are often numerically dominant [72], IS3 elements are more broadly distributed [73]. Host-specialized pathogens such as *Erwinia amylovora* and *Xylella fastidiosa* typically harbor fewer transposable elements, consistent with genome streamlining in stable ecological niches [74, 75]. In contrast, environmentally versatile bacteria such as *Pseudomonas syringae*, *Agrobacterium tumefaciens*, and *Ralstonia solanacearum* contain large numbers of ISs and transposon fragments, reflecting the need for genomic flexibility in variable environments [58]. A notable example is *Xanthomonas oryzae*, where hundreds of IS elements actively reshape genome architecture through gene disruption and rearrangements [58]. This activity contributes to diversification in response to host resistance. Similar patterns are observed in *P. syringae* and *X. campestris*, particularly under agrochemical pressure [76].

Despite being less abundant, transposons play a key role in mobilizing adaptive genes. For example, *Tn5393* carries streptomycin resistance genes (*strA*,* strB*) across multiple phytopathogens [58]. Other transposons mobilize virulence factors, including Type III secretion system effectors such as HopX and PopP2, expanding pathogenic repertoires across species boundaries [58]. Transposon-associated TALEs in *Xanthomonas* further illustrate how these elements contribute to host adaptation by modulating plant gene expression.

**Fig. 4:**
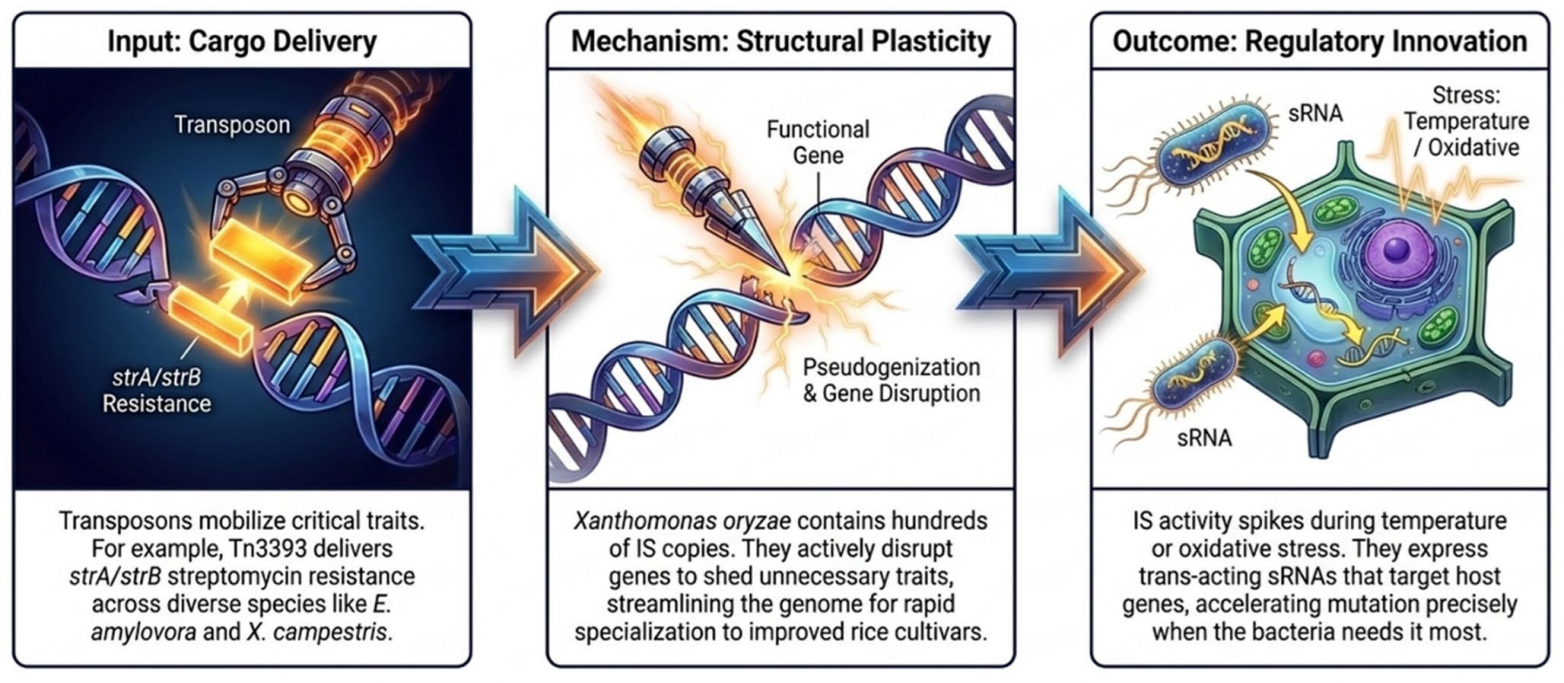
Genome-wide analysis identified thousands of transposable elements across plant pathogenic bacteria. These elements promote host specialization through virulence gene acquisition, genome rearrangements, and gene disruption. Their activity is stress-responsive and can target host signaling pathways. Transposable elements thus represent fundamental drivers of genetic variability and virulence adaptation in phytopathogens

Beyond structural variation, transposable elements also influence gene regulation. Their activity can be modulated by environmental conditions such as temperature and oxidative stress, affecting nearby gene expression, including virulence-associated loci [58]. In addition, some transposons encode small regulatory RNAs that modulate both transposition and host gene expression, reinforcing their role as active regulatory components. Overall, transposons and insertion sequences act as key drivers of genome plasticity in agroecosystems, linking ecological versatility with the capacity for rapid genetic innovation (Fig. 4).

### Integrons as Long-term Evolutionary Devices in Agroecosystem-associated Bacteria: the Xanthomonas Case Study

**Fig. 5:**
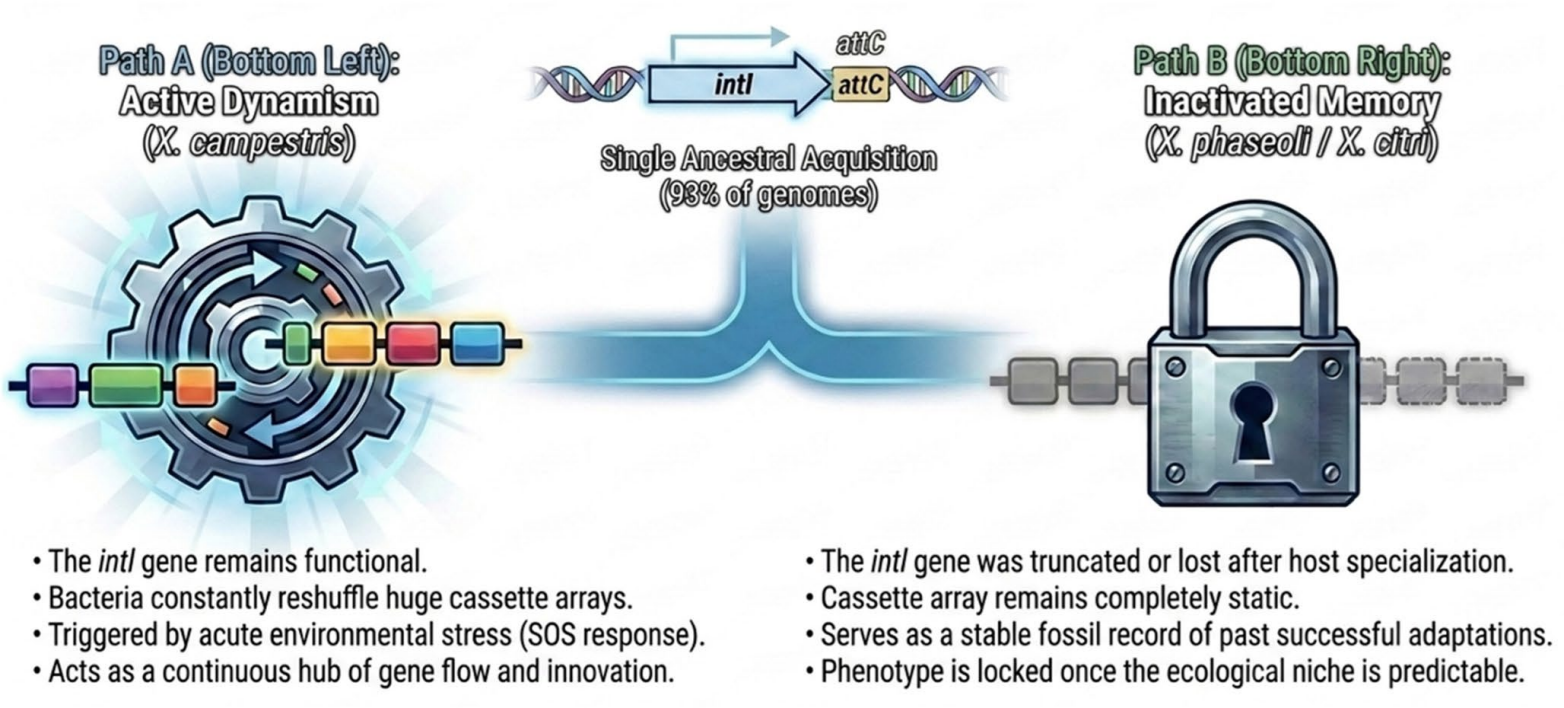
Bifurcating integron evolution in *Xanthomonas*. After a single ancestral acquisition, integrons followed divergent paths: an active, dynamic state in *X. campestris* (high cassette variability) versus an inactivated, stable state in *X. cissicola* and *X. phaseoli* (conserved arrays). Both trajectories provide adaptive cassettes for stress responses and plant interactions, reflecting distinct ecological strategies within the genus

Integrons are sophisticated genetic platforms that capture, rearrange, and express gene cassettes, enabling bacteria to explore genetic novelty under selective pressure. Their activity is often coupled with stress responses such as SOS, making them particularly relevant in agroecosystems characterized by fluctuating nutrients, plant defenses, and agrochemical exposure. While widely studied in clinical antibiotic resistance, integrons are also abundant in soils and plant-associated microbiomes, where most cassette functions remain poorly characterized but likely mediate ecological interactions. The genus *Xanthomonas* is a powerful model for investigating integron evolution in agroecosystems [77]. Analysis of over 700 genomes within this genus shows that integrons are almost everywhere, with integrase genes (*intI*) typically located downstream of *ilvD*. This suggests that they were acquired ancestrally and then transferred by vertical inheritance. Although phylogenetic congruence between *intI* and species relationships supports this, recombination-driven replacement events and phylogenetic incongruence also indicate ongoing horizontal reshaping [63]. Integron functionality varies across lineages. In many species, *intI* is inactivated, whereas in *Xanthomonas campestris* it remains active, with large and highly variable cassette arrays even among closely related strains. In contrast, species such as *X. oryzae* and *X. citri* show loss of integrase activity, suggesting that integrons become dispensable after ecological specialization and stable host adaptation [63]. Despite integrase loss, cassette arrays retain high diversity, encoding thousands of largely uncharacterized genes [78]. When annotated, these genes are associated with secretion, stress tolerance, microbial competition, and phage defense, including restriction–modification and anti-phage systems [79]. Some conserved cassettes, such as *symE*, link integrons to toxin–antitoxin systems and regulatory networks [80]. Horizontal cassette exchange still occurs across species boundaries, particularly from lineages with active integrons such as *X. campestris*, which act as hubs of gene flow in shared agroecosystems [63, 79, 81] (Fig. 5).

Taken together, the *Xanthomonas* case study [77] reveals integrons not as transient genetic curiosities but as long-term evolutionary devices. Their ancestral acquisition, episodic horizontal replacement, lineage-specific inactivation, and residual cassette diversity collectively reflect a history of adaptation to changing agricultural landscapes. Even when integrase activity is lost, cassette arrays preserve a record of past selective pressures, functioning as evolutionary memories embedded within bacterial genomes.

In agroecosystems characterized by recurrent disturbance and strong anthropogenic selection, integrons have provided a mechanism for exploratory evolution -allowing bacteria to sample genetic novelty when needed, and to stabilize successful strategies once ecological niches become predictable. This dual role positions integrons as central, though often overlooked, agents in the evolutionary ecology of plant-associated bacteria.

### PICI and cf-PICI

Phage satellites are a distinct class of mobile genetic elements whose life cycle is strictly dependent on a helper phage. For this reason, they have traditionally been described as “parasites of parasites”. However, accumulating evidence suggests that this definition is overly simplistic and that phage satellites are better understood as partners evolving along a parasitism–symbiosis *continuum*, where antagonistic and mutualistic interactions coexist and shift over evolutionary time [26, 82, 83]. Over the last decade, intensive research has elucidated the molecular mechanisms underlying satellite induction, excision, replication, and packaging, revealing sophisticated strategies by which these elements hijack phage-encoded machinery to ensure their own dissemination [27, 84].

**Fig. 6:**
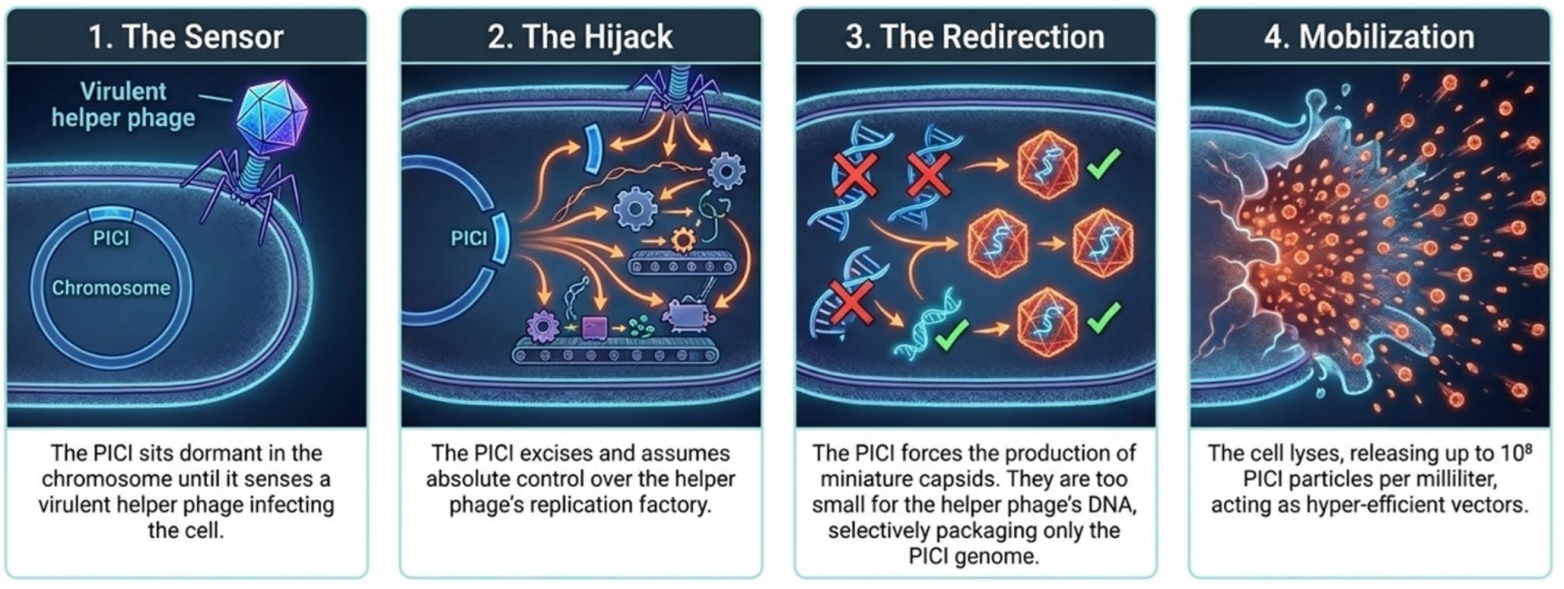
The hijacking life cycle of phage satellites and their evolutionary impact. Phage satellites (e.g., PICIs) parasitize helper phages by redirecting capsid assembly toward satellite packaging, reducing phage virion production while ensuring their own dissemination. Some satellites encode structural components (capsule, tail proteins) for partial self-assembly. This molecular piracy manipulates bacterial traits, from immune evasion to plasmid mobilization and biofilm regulation, making satellites key architects of microbiome evolution in agroecosystems

Early work on phage satellites largely focused on their ability to exploit helper phages for packaging and transfer, as well as on the cargo genes they encode. Many satellites carry toxins, virulence factors, or resistance determinants, prompting investigations into their contribution to bacterial pathogenicity. More recent studies, however, have substantially broadened this view, demonstrating that phage satellites exert far-reaching effects on bacterial evolution and on the dynamics of mobile genetic elements, extending well beyond the direct consequences of their own horizontal transfer [27] (Fig. 6).

The evolutionary relevance of phage satellites became particularly evident with the discovery of *Staphylococcus aureus* pathogenicity islands (SaPIs), which represent the prototypical members of a broader family known as phage-inducible chromosomal islands [26]. PICIs are widespread across bacterial taxa and share a conserved functional architecture that enables them to sense helper phage infection, excise from the chromosome, replicate, and redirect phage assembly toward the production of satellite particles. The identification of additional satellite families, such as the phage-inducible chromosomal island-like elements (PLEs) in *Vibrio cholerae*, further highlighted the ecological importance of these elements [82]. In this system, PLEs actively block the reproduction of the virulent helper phage ICP1, demonstrating that satellites can profoundly shape phage population dynamics and influence host survival during phage epidemics.

Most characterized phage satellites are packaged within capsids composed entirely of helper phage structural proteins. As a result, satellites and phages compete for the same resources, and satellites have evolved multiple strategies to suppress or redirect phage reproduction [83, 85]. A common and highly effective mechanism involves the production of smaller, satellite-sized capsids that selectively package satellite genomes while excluding the larger phage genome [86]. This strategy not only ensures preferential satellite transfer but also dramatically reduces the production of infectious phage particles. Once induced and packaged, PICIs can be mobilized at exceptionally high frequencies, often exceeding 10⁷–10⁸ transducing particles per milliliter under laboratory conditions [87, 88]. Because satellite particles use phage-encoded tails, their host range is dictated by the helper phage, enabling efficient dissemination across susceptible bacterial populations [84].

Integration of satellites into bacterial chromosomes is mediated by satellite-encoded site-specific recombinases, which recognize highly conserved chromosomal attachment sites [84]. These *att*B sites are typically distinct from those targeted by prophages or ICEs, providing a reliable criterion for distinguishing satellites from other integrative mobile elements. This specificity allows satellites to persist stably within host genomes while retaining the capacity for rapid mobilization upon phage induction [89].

Until recently, all known phage satellites were thought to be entirely dependent on helper phages for capsid formation. This view was challenged by the discovery of capsid-forming PICIs (cf-PICIs), a novel and abundant family of satellites capable of producing their own capsids. cf-PICIs encode a set of phage-derived structural and packaging proteins, including major capsid proteins, head maturation proteases, portal proteins, head–tail connectors, and terminize subunits, which are specifically adapted to package the smaller cf-PICI genome into dedicated capsids [90]. Identified across both *γ-Proteobacteria* and *Bacillota*, cf-PICIs represent an unprecedented evolutionary outcome in which satellites have partially emancipated themselves from their helpers, blurring the boundary between phages and their satellites while retaining functional dependence at other stages of the life cycle [91].

Beyond their direct mobilization, PICIs and cf-PICIs play a central role in shaping microbial communities by influencing multiple horizontal gene transfer processes. Satellite activity has been shown to enhance phage-mediated generalized transduction, a process in which fragments of bacterial chromosomes or plasmids are packaged into transducing particles. Rather than representing a costly error, generalized transduction can benefit both bacteria and mobile elements by promoting genetic diversification [26, 92]. By interfering with phage reproduction, satellites protect bacterial populations from phage-driven collapse, increasing the survival of transduced cells and fostering population-level resilience.

PICIs also modulate lysogeny dynamics by reducing the frequency of stable prophage establishment, thereby generating heterogeneous populations composed of lysogenic and non-lysogenic cells [93]. This diversification creates bet-hedging opportunities that enhance adaptive potential under fluctuating environmental conditions. In addition, recent studies have revealed a striking role for PICIs in plasmid evolution and dissemination. The size distribution of conjugative and mobilizable plasmids closely mirrors that of phage genomes and PICI genomes, suggesting that phage- and PICI-mediated transduction acts as a major driver of plasmid mobility in natural populations [94]. Notably, plasmids are often packaged more efficiently by PICI-encoded terminases than by phage terminases, allowing highly efficient plasmid transfer even in the absence of canonical conjugation systems. This mechanism enables plasmid dissemination across species and genus boundaries and imposes evolutionary trade-offs between plasmid size, gene content, and mobility.

Taken together, these findings establish PICIs and cf-PICIs as central players in bacterial evolution, acting not only as selfish mobile elements but also as regulators of phage dynamics, enhancers of horizontal gene transfer, and architects of population-level genetic diversity [84]. Despite their demonstrated importance in clinical and aquatic systems, virtually nothing is currently known about the distribution, diversity, and ecological roles of phage satellites in soil and agricultural environments [95]. Given the intense phage activity, high microbial density, and strong selective pressures characteristic of agroecosystems, it is highly likely that PICIs and cf-PICIs could play a significant yet overlooked role in shaping microbial adaptation and gene flow in these settings. Systematic investigation of phage satellites in soils, plant-associated microbiomes, and agricultural niches therefore represents a critical and timely frontier for understanding bacterial evolution and mobilome dynamics in agroecosystems.

### Genomic Islands

**Fig. 7:**
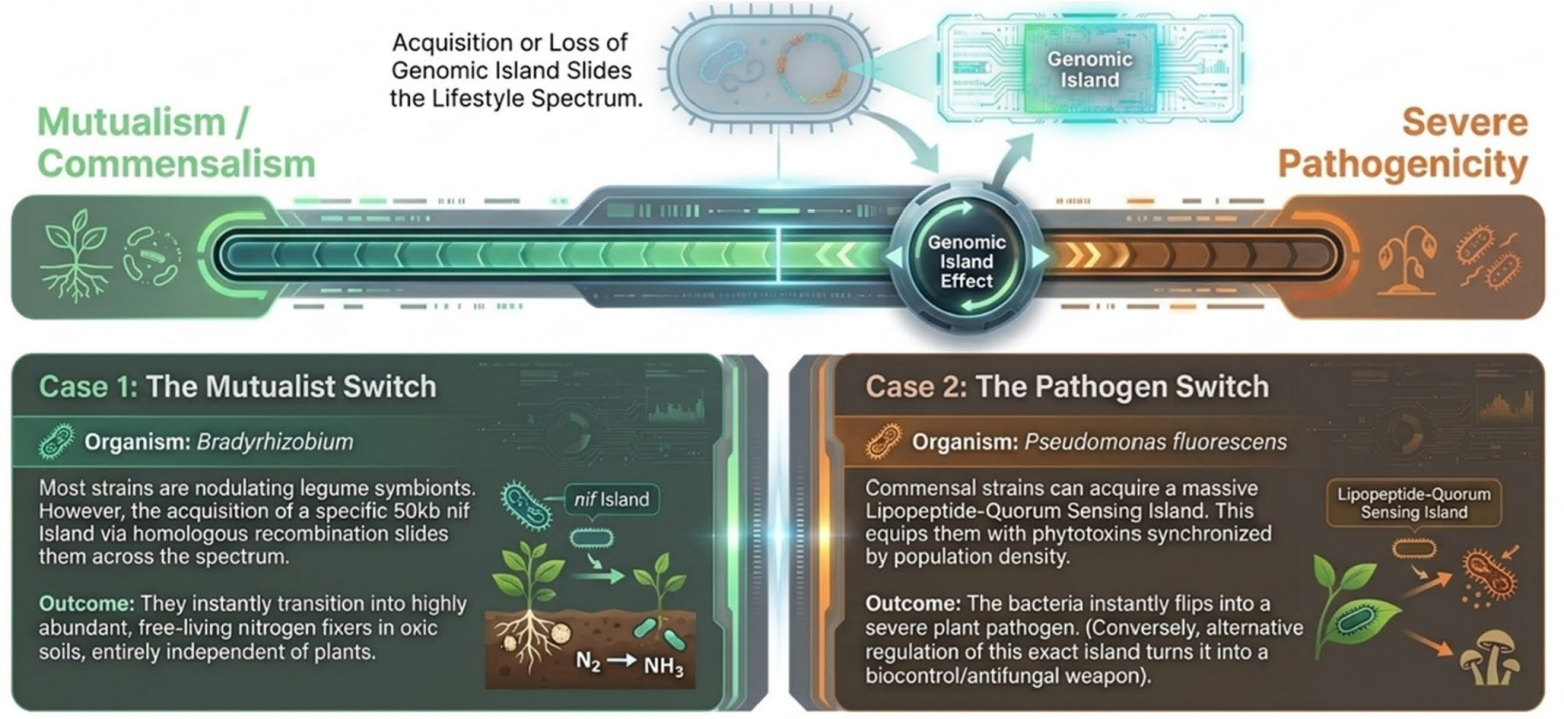
Lifestyle plasticity through genomic island exchange. Genomic islands serve as modular cassettes that bacteria rapidly acquire or discard via homologous recombination, enabling swift transitions between ecological roles (mutualist, commensal, pathogen). Examples in *Bradyrhizobium* and *Pseudomonas* demonstrate how island-encoded traits, from nitrogen fixation to quorum sensing, drive context-dependent functions and convergent evolution. This genomic flexibility underlies bacterial resilience and adaptation to agroecosystem pressures

Genomic islands are key drivers of rapid and reversible lifestyle transitions in plant-associated bacteria, enabling adaptation without extensive core genome change. Their gain and loss allow shifts between mutualism, commensalism, and pathogenicity in response to environmental and agricultural pressures. In *Bradyrhizobium*, genomic islands underpin adaptation to soil environments beyond symbiosis [96]. Although traditionally considered obligate symbionts, many strains are free-living and dominant in soils lacking legumes [97, 98]. This capability is linked to a conserved ~ 50 kb *nif* island encoding nitrogen fixation genes, distinct from classical symbiosis islands. Its phylogenetic incongruence with the core genome indicates horizontal dissemination, likely mediated by homologous recombination at conserved flanking regions rather than classical mobility signatures [91 [99],. This mechanism enables repeated gain and loss, supporting transitions between symbiotic and free-living lifestyles. A similar modular logic is observed in the *Pseudomonas fluorescens* complex [100]. Closely related strains can display contrasting phenotypes, from plant-beneficial to pathogenic, depending on the presence of specific genomic islands. A key example is a large island encoding lipopeptide biosynthesis and quorum sensing, which determines pathogenic behavior. Its distribution reflects repeated gain and loss via homologous recombination, rather than classical mobile element activity. Importantly, the ecological outcome of such islands depends on regulatory context. In some strains, the same lipopeptide island supports antifungal activity and biocontrol rather than pathogenicity, illustrating functional plasticity [88]. At the population level, genomic islands interact epistatically with other loci, forming combinations that define ecological niches and constrain alternative lifestyles. Overall, genomic islands act as modular evolutionary units that enable rapid ecological shifts, supporting nitrogen fixation, virulence, or microbial interactions, while maintaining core genome stability. In agroecosystems, where communities are continuously reshaped by management and environmental pressures, this modularity represents a major mechanism of bacterial adaptation and innovation [101] (Fig. 7).

## MGEs Beyond Model Organisms: Limits of Extrapolation and Emerging Approaches in Agroecosystem Microbiomes

The current mechanistic understanding of mobile genetic elements derives from a relatively small number of model organisms, including well-characterized representatives of species such as *Escherichia coli*,* Pseudomonas fluorescens*, and *Bacillus subtilis* [30]. These systems have been instrumental in elucidating the molecular basis of horizontal gene transfer, including plasmid conjugation, phage infection cycles, transposition, and the regulation of integrative and conjugative elements. However, agricultural microbiomes are dominated by highly diverse and often uncultured taxa. This raises fundamental questions about the extent to which insights derived from model systems can be generalized to complex agroecosystems [102]. A key limitation of model-based knowledge lies in the ecological simplification inherent to laboratory systems. Most experimental frameworks rely on homogeneous, well-mixed environments and a limited number of interacting strains, conditions that contrast sharply with the spatial heterogeneity and physicochemical complexity of soils [103]. In natural agroecosystems, microbial cells are embedded in structured matrices characterized by microscale gradients of oxygen, nutrients, moisture, and pH. These features can strongly constrain cell-to-cell contact, diffusion of signaling molecules, and ultimately the frequency and directionality of MGE transfer. As a result, transfer rates and host ranges measured in vitro assays may substantially overestimate the in situ effective mobility of MGEs [104]. Another major challenge concerns host range and compatibility. Many MGEs characterized in model organisms display broad host ranges under laboratory conditions, yet their persistence in natural communities depends on complex ecological and evolutionary filters. These include restriction–modification systems, CRISPR-Cas immunity, and other defense mechanisms that can limit successful establishment in new hosts. Such barriers are widespread in environmental bacteria, contributing to shaping the structure of gene flow networks in soils. Consequently, the apparent promiscuity of MGEs observed in model systems may not translate directly to field conditions, where gene exchange is likely restricted to phylogenetically or ecologically compatible populations [105]. Importantly, the vast majority of soil and plant-associated bacteria remain uncultured or poorly characterized, limiting direct experimental validation of MGE dynamics. This “uncultured majority” represents a substantial blind spot in our understanding of mobilomes. Recent advances in metagenomics and long-read sequencing are beginning to overcome these limitations by enabling the reconstruction of plasmids, prophages, and genomic islands directly from environmental samples. These approaches have revealed an unexpectedly high diversity of MGEs, many of which lack homologs in existing databases, suggesting that current model systems capture only a fraction of mobilome diversity [2, 104]. Despite these challenges, new methodological frameworks are emerging to bridge the gap between model systems and complex microbiomes [106, 107]. High-resolution metagenomics, coupled with proximity ligation techniques (e.g., Hi-C), now allows the association of MGEs with their host genomes in situ, providing insights into host range and transfer networks within natural communities. Similarly, single-cell genomics and microfluidic platforms offer the possibility to directly observe MGE transfer under controlled but ecologically relevant conditions, capturing dynamics that are otherwise inaccessible in bulk experiments. Experimental evolution in soil microcosms and synthetic communities further enables the study of MGE persistence, fitness costs, and ecological impacts across multiple taxa simultaneously. Nevertheless, important limitations remain. Many of these approaches provide correlative rather than causal insights, making it difficult to disentangle active transfer from historical acquisition. Additionally, biases in DNA extraction, assembly, and binning can affect the detection and reconstruction of MGEs, particularly for low-abundance elements or highly repetitive sequences. Functional validation also remains a bottleneck, as genetic manipulation tools are often unavailable for non-model organisms, restricting our ability to directly test the ecological roles of specific MGEs. Taken together, these considerations highlight that while model organisms have provided a foundational understanding of MGE biology, extrapolation to agroecosystem microbiomes must be approached with caution. Rather than assuming universality, it is more appropriate to view model-derived mechanisms as conceptual frameworks that require validation and contextualization in complex environments. Future progress will depend on integrating cultivation-independent genomics with experimental ecology and developing new tools for manipulating and tracking MGEs in diverse bacterial hosts. Such efforts are essential for achieving a predictive understanding of how MGEs shape microbial evolution and ecosystem functioning in agricultural soils.

## Implications and Future Directions

In agroecosystems, mobile genetic elements (MGEs) represent a central component of microbial evolutionary dynamics, mediating bacterial response to fluctuating environmental conditions, management practices, and host-associated selective pressures [2]. Rather than representing exceptional or disruptive features, MGEs form an integral component of microbial genome architecture, supporting rapid functional turnover while maintaining relatively stable core genome [108]. Agricultural soils, characterized by recurrent disturbance, spatial heterogeneity, and strong anthropogenic inputs, provide ideal contexts in which mobilome-driven processes are likely to play a prominent role. In these systems, plasmids, integrative and conjugative elements, transposons, integrons, phage-related elements, and defensive islands collectively shape microbial performance and ecosystem function [109]. From this ecological perspective, antibiotic resistance can be interpreted as one outcome of a broader set of adapative responses mediated by MGEs rather than an isolated phenomenon. The same mobile platforms responsible for resistance gene dissemination also mobilize genes associated with nutrient acquisition, stress tolerance, plant colonization, microbial antagonism, and modulation of host responses [30]. Fertilizers, pesticides, plant-derived metabolites, and abiotic stressors such as drought or salinity act as powerful co-selective forces, influencing the composition and activity of the mobilome [31]. Consequently, MGEs act as environmentally responsive reservoirs of genetic variability, facilitating rapid adjustment of to changing conditions. Importantly, this genetic plasticity is not unconstrained: agroecosystem bacteria also harbor diverse defense islands, including restriction–modification systems, CRISPR–Cas arrays, and other anti-MGE mechanisms, which limit uncontrolled horizontal gene transfer [108]. These systems impose a selective filter on gene exchange, maintaining genome integrity while still permitting adaptive innovation. The evolutionary impact of MGEs in agroecosystems is strongly shaped by microbial lifestyle and niche specialization. Generalist soil and rhizosphere bacteria tend to maintain active and diverse mobilomes that facilitate ongoing adaptation, whereas highly specialized lineages often exhibit progressive streamlining or inactivation of mobile elements, retaining only remnants of past genetic exchange [110]. This highlights MGEs as drivers of both short-term gene flow and longer-term evolutionary trajectories influencing soil fertility and crop productivity. Recognizing agroecosystem bacteria as modular evolutionary entities, composed of a relatively stable core genome and a fluid, environment-sensitive mobilome, has important implications for microbiome monitoring and management. Approaches focused solely on taxonomic composition risk overlooking the genetic processes that directly control microbial function. Incorporating mobilome-level analyses into agroecosystem surveillance offers a more predictive framework for assessing soil health and forecasting microbiome responses to management interventions [109]. This perspective also opens new opportunities for sustainable agriculture. MGEs could be harnessed to facilitate the controlled dissemination of traits that enhance nutrient cycling, disease suppression, stress tolerance, or plant growth promotion within defined ecological boundaries. By enabling the spread of functions such as nitrogen fixation, phosphorus solubilization, or xenobiotic degradation, MGEs may contribute to restoring degraded soils and improving long-term ecosystem resilience. However, such strategies require a detailed understanding of the ecological constraints governing MGE transfer and persistence to avoid unintended gene flow or disruption of native communities [111].

Despite these advances, several fundamental questions remain unresolved. A primary challenge is to quantify MGE transfer rates under realistic agroecosystem conditions. While laboratory studies have identified key determinants of horizontal gene transfer, in situ transfer is highly context-dependent and influenced by microbial density, nutrient availability, environmental stress, and spatial organization. Similarly, it remains unclear under which ecological conditions different classes of MGEs are favored. Plasmids, integrative elements, and phage-mediated systems differ in transmission modes, host range, and fitness costs, but the selective pressures governing their relative success in soils and plant-associated microbiomes are still poorly defined. Another major gap concerns the role of physical structure. Soil heterogeneity creates microscale niches that may act as hotspots or barriers for gene transfer, particularly in the rhizosphere, where microbial activity is intensified. Likewise, plant-associated compartments such as the rhizosphere, endosphere, and phyllosphere differ in connectivity and population density, potentially shaping distinct mobilome dynamics. The extent to which host plants actively influence MGE transfer through exudation patterns, immune signaling, or microbiome filtering remains largely unexplored.

Finally, a key frontier lies in integrating empirical observations with predictive frameworks. Advances in long-read metagenomics, single-cell approaches, and microcosm experiments are beginning to elucidate the structure and activity of mobilomes in situ. Linking these data with ecological modeling and machine-learning approaches may enable more robust predictions of mobilome dynamics across cropping systems, identify practices that favor beneficial gene flow, and anticipate unintended consequences of agricultural intensification [9].

## Data Availability

No datasets were generated or analysed during the current study.

## Declarations

### Generative AI and AI-assisted technologies in the manuscript preparation process

During the preparation of this work the author(s) used NotebookLM in order to draw the concept of some figures. After using this tool/service, the author(s) reviewed and edited the content as needed and take(s) full responsibility for the content of the published article.

## References

[1] Benler S, Koonin EV (2022) Recruitment of Mobile Genetic Elements for Diverse Cellular Functions in Prokaryotes. Front Mol Biosci 9:821197. 10.3389/fmolb.2022.821197 35402511 10.3389/fmolb.2022.821197 PMC8987985

[2] Tokuda M, Shintani M (2024) Microbial evolution through horizontal gene transfer by mobile genetic elements. Microb Biotechnol 17:e14408. 10.1111/1751-7915.14408 38226780 10.1111/1751-7915.14408 PMC10832538

[3] Rodríguez-Beltrán J, DelaFuente J, León-Sampedro R et al (2021) Beyond horizontal gene transfer: the role of plasmids in bacterial evolution. Nat Rev Microbiol 19:347–359. 10.1038/s41579-020-00497-1 33469168 10.1038/s41579-020-00497-1

[4] Lipszyc A, Szuplewska M, Bartosik D (2022) How do transposable elements activate expression of transcriptionally silent antibiotic resistance genes? Int J Mol Sci 23:8063. 10.3390/ijms23158063 35897639 10.3390/ijms23158063 PMC9330008

[5] Loot C, Millot GA, Richard E et al (2024) Integron cassettes integrate into bacterial genomes via widespread non-classical attG sites. Nat Microbiol 9:228–240. 10.1038/s41564-023-01548-y 38172619 10.1038/s41564-023-01548-y

[6] Singh H, Pandya S, Jasani S et al (2025) Integrons: the hidden architects of bacterial adaptation, evolution, and the challenges of antimicrobial resistance. Antonie Van Leeuwenhoek. 10.1007/s10482-025-02103-x 40506592 10.1007/s10482-025-02103-x

[7] Zhang Y, Guo Y, Qiu T et al (2022) Bacteriophages: underestimated vehicles of antibiotic resistance genes in the soil. Front Microbiol 13:936267. 10.3389/fmicb.2022.936267 35992716 10.3389/fmicb.2022.936267 PMC9386270

[8] Gonçalves OS, De Assis JCS, Santana MF (2022) Breaking the ICE: an easy workflow for identifying and analyzing integrative and conjugative elements in bacterial genomes. Funct Integr Genomics 22:1139–1145. 10.1007/s10142-022-00903-2 36149586 10.1007/s10142-022-00903-2

[9] Weisberg AJ, Chang JH (2023) Mobile Genetic Element Flexibility as an Underlying Principle to Bacterial Evolution. Annu Rev Microbiol 77:603–624. 10.1146/annurev-micro-032521-022006 37437216 10.1146/annurev-micro-032521-022006

[10] Lang AS, Buchan A, Burrus V (2025) Interactions and evolutionary relationships among bacterial mobile genetic elements. Nat Rev Microbiol 23:423–438. 10.1038/s41579-025-01157-y 40069292 10.1038/s41579-025-01157-y

[11] Brito IL (2021) Examining horizontal gene transfer in microbial communities. Nat Rev Microbiol 19:442–453. 10.1038/s41579-021-00534-7 33846600 10.1038/s41579-021-00534-7

[12] Paul D, Verma J, Banerjee A et al (2022) Antimicrobial Resistance Traits and Resistance Mechanisms in Bacterial Pathogens. In: Kumar V, Shriram V, Paul A, Thakur M (eds) Antimicrobial Resistance. Springer Nature Singapore, Singapore, pp 1–27

[13] Arnold BJ, Huang I-T, Hanage WP (2022) Horizontal gene transfer and adaptive evolution in bacteria. Nat Rev Microbiol 20:206–218. 10.1038/s41579-021-00650-4 34773098 10.1038/s41579-021-00650-4

[14] Gasparotto E, Burattin FV, Di Gioia V et al (2023) Transposable Elements Co-Option in Genome Evolution and Gene Regulation. IJMS 24:2610. 10.3390/ijms24032610 36768929 10.3390/ijms24032610 PMC9917352

[15] Samuel B, Mittelman K, Croitoru SY et al (2024) Diverse anti-defence systems are encoded in the leading region of plasmids. Nature 635:186–192. 10.1038/s41586-024-07994-w 39385022 10.1038/s41586-024-07994-w PMC11541004

[16] Vale FF, Lehours P, Yamaoka Y (2022) Editorial: The Role of Mobile Genetic Elements in Bacterial Evolution and Their Adaptability. Front Microbiol 13:849667. 10.3389/fmicb.2022.849667 35265063 10.3389/fmicb.2022.849667 PMC8899501

[17] Whelan FJ, Hall RJ, McInerney JO (2021) Evidence for selection in the abundant accessory gene content of a prokaryote pangenome. Mol Biol Evol 38:3697–3708. 10.1093/molbev/msab139 33963386 10.1093/molbev/msab139 PMC8382901

[18] Rosconi F, Rudmann E, Li J et al (2022) A bacterial pan-genome makes gene essentiality strain-dependent and evolvable. Nat Microbiol 7:1580–1592. 10.1038/s41564-022-01208-7 36097170 10.1038/s41564-022-01208-7 PMC9519441

[19] Xiong C, Lu Y (2022) Microbiomes in agroecosystem: Diversity, function and assembly mechanisms. Environ Microbiol Rep 14:833–849. 10.1111/1758-2229.13126 36184075 10.1111/1758-2229.13126

[20] Daza BXD, Mendoza AJD, Zambrano JJZ et al (2024) Effects of Soil Contaminants on Soil Microbiome. In: Aransiola SA, Atta HI, Maddela NR (eds) Soil Microbiome in Green Technology Sustainability. Springer Nature Switzerland, Cham, pp 183–199

[21] Khan MM, Bhatt P (2023) Editorial: Environmental pollutants in agroecosystem: toxicity, mechanism, and remediation. Front Plant Sci 14:1208405. 10.3389/fpls.2023.1208405 37351214 10.3389/fpls.2023.1208405 PMC10282987

[22] Hartmann M, Six J (2022) Soil structure and microbiome functions in agroecosystems. Nat Rev Earth Environ 4:4–18. 10.1038/s43017-022-00366-w

[23] Huang H, Lin L, Bu F et al (2022) Reductive Stress Boosts the Horizontal Transfer of Plasmid-Borne Antibiotic Resistance Genes: The Neglected Side of the Intracellular Redox Spectrum. Environ Sci Technol 56:15594–15606. 10.1021/acs.est.2c04276 36322896 10.1021/acs.est.2c04276

[24] Colombi E, Bertels F, Doulcier G et al (2024) Rapid dissemination of host metabolism–manipulating genes via integrative and conjugative elements. Proc Natl Acad Sci USA 121:e2309263121. 10.1073/pnas.2309263121 38457521 10.1073/pnas.2309263121 PMC10945833

[25] Singh H, Pandya S, Jasani S et al (2025) Integrons: the hidden architects of bacterial adaptation, evolution, and the challenges of antimicrobial resistance. Antonie Van Leeuwenhoek 118:90. 10.1007/s10482-025-02103-x 40506592 10.1007/s10482-025-02103-x

[26] Ibarra-Chávez R, Brady A, Chen J et al (2022) Phage-inducible chromosomal islands promote genetic variability by blocking phage reproduction and protecting transductants from phage lysis. PLoS Genet 18:e1010146. 10.1371/journal.pgen.1010146 35344558 10.1371/journal.pgen.1010146 PMC8989297

[27] Penadés JR, Seed KD, Chen J et al (2025) Genetics, ecology and evolution of phage satellites. Nat Rev Microbiol 23:410–422. 10.1038/s41579-025-01156-z 40148600 10.1038/s41579-025-01156-z

[28] Banerjee S, Van Der Heijden MGA (2023) Soil microbiomes and one health. Nat Rev Microbiol 21:6–20. 10.1038/s41579-022-00779-w 35999468 10.1038/s41579-022-00779-w

[29] Brennan FP, Alsanius BW, Allende A et al (2022) Harnessing agricultural microbiomes for human pathogen control. ISME Commun 2:44. 10.1038/s43705-022-00127-2 37938739 10.1038/s43705-022-00127-2 PMC9723689

[30] Rajabal V, Ghaly TM, Egidi E et al (2024) Exploring the role of mobile genetic elements in shaping plant–bacterial interactions for sustainable agriculture and ecosystem health. Plants People Planet 6:408–420. 10.1002/ppp3.10448

[31] Kumavath R, Gupta P, Tatta ER et al (2025) Unraveling the role of mobile genetic elements in antibiotic resistance transmission and defense strategies in bacteria. Frontiers in Systems Biology 5:1557413. 10.3389/fsysb.2025.1557413 40810119 10.3389/fsysb.2025.1557413 PMC12342005

[32] Li Y, Li R, Hou J et al (2024) Mobile genetic elements affect the dissemination of antibiotic resistance genes (ARGs) of clinical importance in the environment. Environ Res 243:117801. 10.1016/j.envres.2023.117801 38043895 10.1016/j.envres.2023.117801

[33] Zhang Y, Kitazumi A, Liao Y-T et al (2023) Metagenomic investigation reveals bacteriophage-mediated horizontal transfer of antibiotic resistance genes in microbial communities of an organic agricultural ecosystem. Microbiol Spectr 11:e00226-23. 10.1128/spectrum.00226-23 37754684 10.1128/spectrum.00226-23 PMC10581182

[34] Aworh MK, Lawal OU, Egyir B, Hendriksen RS (2025) In silico genomic insights into bacteriophages infecting ESBL-producing Escherichia coli from human, animal, and environmental sources. BMC Microbiol 25:200. 10.1186/s12866-025-03913-9 40200154 10.1186/s12866-025-03913-9 PMC11978167

[35] Visca A, Di Gregorio L, Clagnan E, Bevivino A (2024) Sustainable strategies: nature-based solutions to tackle antibiotic resistance gene proliferation and improve agricultural productivity and soil quality. Environ Res 248:118395. 10.1016/j.envres.2024.118395 38307185 10.1016/j.envres.2024.118395

[36] Visca A, Rauseo J, Spataro F et al (2022) Antibiotics and antibiotic resistance genes in anaerobic digesters and predicted concentrations in agroecosystems. J Environ Manage 301:113891. 10.1016/j.jenvman.2021.113891 34731939 10.1016/j.jenvman.2021.113891

[37] Lu W, Wang M, Wu J et al (2020) Spread of chloramphenicol and tetracycline resistance genes by plasmid mobilization in agricultural soil. Environ Pollut 260:113998. 10.1016/j.envpol.2020.113998 31991360 10.1016/j.envpol.2020.113998

[38] Klümper U, Riber L, Dechesne A et al (2015) Broad host range plasmids can invade an unexpectedly diverse fraction of a soil bacterial community. ISME J 9:934–945. 10.1038/ismej.2014.191 25333461 10.1038/ismej.2014.191 PMC4817699

[39] Heuer H, Smalla K (2012) Plasmids foster diversification and adaptation of bacterial populations in soil. FEMS Microbiol Rev 36:1083–1104. 10.1111/j.1574-6976.2012.00337.x 22393901 10.1111/j.1574-6976.2012.00337.x

[40] Niault T, Van Houte S, Westra E, Swarts DC (2025) Evolution and ecology of anti-defence systems in phages and plasmids. Curr Biol 35:R32–R44. 10.1016/j.cub.2024.11.033 39765217 10.1016/j.cub.2024.11.033

[41] Pal R, Poddar BJ, D. Pandit P et al (2026) Pan-genome analysis of *Morganella morganii* reveals niche-specific selection of functional traits: friend or foe? Arch Microbiol 208:40. 10.1007/s00203-025-04566-y 10.1007/s00203-025-04566-y 41313384

[42] Bhosale S, Saha S, Patil N (2025) Insights on plasmids and their role in evolution of *Xanthomonas*. Australas Plant Pathol 54:241–252. 10.1007/s13313-025-01048-z

[43] Norberg P, Bergström M, Jethava V et al (2011) The IncP-1 plasmid backbone adapts to different host bacterial species and evolves through homologous recombination. Nat Commun 2:268. 10.1038/ncomms1267 21468020 10.1038/ncomms1267 PMC3104523

[44] Shintani M, Nour E, Elsayed T et al (2020) Plant species-dependent increased abundance and diversity of IncP-1 plasmids in the rhizosphere: new insights into their role and ecology. Front Microbiol 11:590776. 10.3389/fmicb.2020.590776 33329469 10.3389/fmicb.2020.590776 PMC7728920

[45] Cotta SR, Dias ACF, Mendes R, Andreote FD (2025) Role of horizontal gene transfer and cooperation in rhizosphere microbiome assembly. Braz J Microbiol 56:225–236. 10.1007/s42770-024-01583-9 39730778 10.1007/s42770-024-01583-9 PMC11885732

[46] Hauschild K, Suzuki M, Wolters B et al (2025) The transferable resistome of biosolids—plasmid sequencing reveals carriage of clinically relevant antibiotic resistance genes. mBio 16:e02068-25. 10.1128/mbio.02068-25 41104936 10.1128/mbio.02068-25 PMC12607642

[47] Werner J, Nour E, Bunk B et al (2020) PromA plasmids are instrumental in the dissemination of linuron catabolic genes between different genera. Front Microbiol 11:149. 10.3389/fmicb.2020.00149 32132980 10.3389/fmicb.2020.00149 PMC7039861

[48] Dennis JJ (2005) The evolution of IncP catabolic plasmids. Curr Opin Biotechnol 16:291–298. 10.1016/j.copbio.2005.04.002 15961030 10.1016/j.copbio.2005.04.002

[49] Jauregi L, González A, Garbisu C, Epelde L (2023) Organic amendment treatments for antimicrobial resistance and mobile element genes risk reduction in soil-crop systems. Sci Rep 13:863. 10.1038/s41598-023-27840-9 36650207 10.1038/s41598-023-27840-9 PMC9845208

[50] Meng M, Li Y, Yao H (2022) Plasmid-mediated transfer of antibiotic resistance genes in soil. Antibiotics (Basel) 11:525. 10.3390/antibiotics11040525 35453275 10.3390/antibiotics11040525 PMC9024699

[51] Gillieatt BF, Thai M, Cain AK et al (2025) The role of heavy metals in the co-selection of plasmid-borne metal and antibiotic resistance genes from industrially contaminated sediments

[52] Liu Z, Zhao Q, Xu C, Song H (2024) Compensatory evolution of chromosomes and plasmids counteracts the plasmid fitness cost. Ecol Evol 14:e70121. 10.1002/ece3.70121 39170056 10.1002/ece3.70121 PMC11336059

[53] Curtsinger HD, Martínez-Absalón S, Liu Y, Lopatkin AJ (2025) The metabolic burden associated with plasmid acquisition: an assessment of the unrecognized benefits to host cells. Bioessays 47:2400164. 10.1002/bies.202400164 10.1002/bies.202400164 PMC12126754 39529437

[54] Newbury A, Dawson B, Klümper U et al (2022) Fitness effects of plasmids shape the structure of bacteria–plasmid interaction networks. Proc Natl Acad Sci U S A 119:e2118361119. 10.1073/pnas.2118361119 35613058 10.1073/pnas.2118361119 PMC9295774

[55] Domingues CPF, Rebelo JS, Monteiro F et al (2022) Harmful behaviour through plasmid transfer: a successful evolutionary strategy of bacteria harbouring conjugative plasmids. Philos Trans R Soc Lond B Biol Sci 377:20200473. 10.1098/rstb.2020.0473 34839709 10.1098/rstb.2020.0473 PMC8628071

[56] Rajer F, Sandegren L (2022) The Role of Antibiotic Resistance Genes in the Fitness Cost of Multiresistance Plasmids. mBio 13:e03552–e03521. 10.1128/mbio.03552-21 35038907 10.1128/mbio.03552-21 PMC8764527

[57] Carraro N, Burrus V (2015) The dualistic nature of integrative and conjugative elements. Mob Genet Elem 5:98–102. 10.1080/2159256X.2015.1102796 10.1080/2159256X.2015.1102796 PMC4755238 26942046

[58] De Assis JCS, Gonçalves OS, Fernandes AS et al (2022) Genomic analysis reveals the role of integrative and conjugative elements in plant pathogenic bacteria. Mob DNA 13:19. 10.1186/s13100-022-00275-1 35962419 10.1186/s13100-022-00275-1 PMC9373382

[59] Johnson CM, Grossman AD (2015) Integrative and Conjugative Elements (ICEs): What They Do and How They Work. Annu Rev Genet 49:577–601. 10.1146/annurev-genet-112414-055018 26473380 10.1146/annurev-genet-112414-055018 PMC5180612

[60] Colombi E, Straub C, Künzel S et al (2017) Evolution of copper resistance in the kiwifruit pathogen *P seudomonas syringae* pv. *actinidiae* through acquisition of integrative conjugative elements and plasmids. Environ Microbiol 19:819–832. 10.1111/1462-2920.13662 28063194 10.1111/1462-2920.13662

[61] Puttilli MR, Danzi D, Correia C et al (2022) Plant Signals Anticipate the Induction of the Type III Secretion System in Pseudomonas syringae pv. *actinidiae*, Facilitating Efficient Temperature-Dependent Effector Translocation. Microbiol Spectr 10:e02073–e02022. 10.1128/spectrum.02073-22 36287008 10.1128/spectrum.02073-22 PMC9770001

[62] Yu H, Li Y, Han W et al (2024) Pan-evolutionary and regulatory genome architecture delineated by an integrated macro- and microsynteny approach. Nat Protoc 19:1623–1678. 10.1038/s41596-024-00966-4 38514839 10.1038/s41596-024-00966-4

[63] Watanabe Y, Ishiga Y, Sakata N (2025) The Role of Genomic Islands in the Pathogenicity and Evolution of Plant-Pathogenic Gammaproteobacteria. Microorganisms 13:1803. 10.3390/microorganisms13081803 40871307 10.3390/microorganisms13081803 PMC12388829

[64] Wardell GE, Hynes MF, Young PJ, Harrison E (2022) Why are rhizobial symbiosis genes mobile? Philosophical Trans Royal Soc B 377:20200471. 10.1098/rstb.2020.0471 10.1098/rstb.2020.0471 PMC8628070 34839705

[65] Han K, Li Y, Zhang Z et al (2023) Comparative genome analysis of Sesbania cannabina-nodulating Rhizobium spp. revealing the symbiotic and transferrable characteristics of symbiosis plasmids. Microb Genomics 9. 10.1099/mgen.0.001004 10.1099/mgen.0.001004 PMC10272884 37133904

[66] Provorov NA, Andronov EE, Kimeklis AK et al (2022) Microevolution, speciation and macroevolution in rhizobia: Genomic mechanisms and selective patterns. Front Plant Sci 13:1026943. 10.3389/fpls.2022.1026943 36388581 10.3389/fpls.2022.1026943 PMC9640933

[67] Choufa C, Tidjani A-R, Gauthier A et al (2022) Prevalence and mobility of integrative and conjugative elements within a Streptomyces natural population. Front Microbiol 13:970179. 10.3389/fmicb.2022.970179 36177458 10.3389/fmicb.2022.970179 PMC9513070

[68] Mavrodi OV, McWilliams JR, Peter JO et al (2021) Root Exudates Alter the Expression of Diverse Metabolic, Transport, Regulatory, and Stress Response Genes in Rhizosphere Pseudomonas. Front Microbiol 12:651282. 10.3389/fmicb.2021.651282 33936009 10.3389/fmicb.2021.651282 PMC8079746

[69] Liu S, Jiao J, Tian C-F (2023) Adaptive evolution of rhizobial symbiosis beyond horizontal gene transfer: from genome innovation to regulation reconstruction. Genes (Basel) 14:274. 10.3390/genes14020274 36833201 10.3390/genes14020274 PMC9957244

[70] Colombi E, Perry BJ, Sullivan JT et al (2021) Comparative analysis of integrative and conjugative mobile genetic elements in the genus Mesorhizobium. Microb Genomics 7. 10.1099/mgen.0.000657 10.1099/mgen.0.000657 PMC8627217 34605762

[71] Fernandes AS, Campos KF, De Assis JCS et al (2024) Investigating the impact of insertion sequences and transposons in the genomes of the most significant phytopathogenic bacteria. Microb Genomics. 10.1099/mgen.0.001219 10.1099/mgen.0.001219 PMC11092175 38568199

[72] Onstead J, Zhang Z, Huo J et al (2024) Investigating How Genomic Contexts Impact IS5 Transposition Within the Escherichia coli Genome. Microorganisms 12:2600. 10.3390/microorganisms12122600 39770802 10.3390/microorganisms12122600 PMC11677980

[73] Tempel S, Bedo J, Talla E (2022) From a large-scale genomic analysis of insertion sequences to insights into their regulatory roles in prokaryotes. BMC Genomics 23:451. 10.1186/s12864-022-08678-3 35725380 10.1186/s12864-022-08678-3 PMC9208149

[74] López Díaz C, Ayhan DH, Rodríguez López A et al (2025) Transposons and accessory genes drive adaptation in a clonally evolving fungal pathogen. Nat Commun 16:6982. 10.1038/s41467-025-62213-y 40739135 10.1038/s41467-025-62213-y PMC12310939

[75] Lajoie G, Parfrey LW (2022) Beyond specialization: re-examining routes of host influence on symbiont evolution. Trends Ecol Evol 37:590–598. 10.1016/j.tree.2022.03.006 35466020 10.1016/j.tree.2022.03.006

[76] Assis RAB, Varani AM, Sagawa CHD et al (2021) A comparative genomic analysis of Xanthomonas arboricola pv. juglandis strains reveal hallmarks of mobile genetic elements in the adaptation and accelerated evolution of virulence. Genomics 113:2513–2525. 10.1016/j.ygeno.2021.06.003 34089784 10.1016/j.ygeno.2021.06.003

[77] Colombi E, Ghaly TM, Rajabal V et al (2025) Adaptative and ancient co-evolution of integrons with Xanthomonas genomes. Microbial Genomics 11:. 10.1099/mgen.0.001503 10.1099/mgen.0.001503 PMC12444791 40961345

[78] Gillings MR, Holley MP, Stokes HW, Holmes AJ (2005) Integrons in *Xanthomonas*: a source of species genome diversity. Proc Natl Acad Sci U S A 102:4419–4424. 10.1073/pnas.0406620102 15755815 10.1073/pnas.0406620102 PMC555480

[79] Ghaly TM, Rajabal V, Penesyan A et al (2023) Functional enrichment of integrons: facilitators of antimicrobial resistance and niche adaptation. iScience 26:108301. 10.1016/j.isci.2023.108301 38026211 10.1016/j.isci.2023.108301 PMC10661359

[80] Darracq B, Littner E, Brunie M et al (2024) Sedentary chromosomal integrons as biobanks of bacterial anti-phage defence systems 10.1126/science.ads0768 40339013

[81] Ghaly TM, Tetu SG, Gillings MR (2021) Predicting the taxonomic and environmental sources of integron gene cassettes using structural and sequence homology of attC sites. Commun Biol 4:946. 10.1038/s42003-021-02489-0 34373573 10.1038/s42003-021-02489-0 PMC8352920

[82] Fillol-Salom A, Martínez-Rubio R, Abdulrahman RF et al (2018) Phage-inducible chromosomal islands are ubiquitous within the bacterial universe. ISME J 12:2114–2128. 10.1038/s41396-018-0156-3 29875435 10.1038/s41396-018-0156-3 PMC6092414

[83] Ibarra-Chávez R, Hansen MF, Pinilla-Redondo R et al (2021) Phage satellites and their emerging applications in biotechnology. FEMS Microbiol Rev 45:fuab031. 10.1093/femsre/fuab031 34104956 10.1093/femsre/fuab031 PMC8632786

[84] Alqurainy N, Miguel-Romero L, Moura De Sousa J et al (2023) A widespread family of phage-inducible chromosomal islands only steals bacteriophage tails to spread in nature. Cell Host Microbe 31:69–82. 10.1016/j.chom.2022.12.001 36596306 10.1016/j.chom.2022.12.001

[85] Ojiogu AD, Patkowski JB, Kuang X et al (2025) Capsid redirection mechanism of the *Staphylococcus aureus* pathogenicity island SaPIpT1028. Phil Trans R Soc B 380:20240075. 10.1098/rstb.2024.0075 40904114 10.1098/rstb.2024.0075 PMC12409344

[86] Boyd CM, Subramanian S, Dunham DT et al (2024) A Vibrio cholerae viral satellite maximizes its spread and inhibits phage by remodeling hijacked phage coat proteins into small capsids. eLife 12:RP87611. 10.7554/eLife.87611 38206122 10.7554/eLife.87611 PMC10945586

[87] Alqurainy N, Miguel-Romero L, De Moura J et al (2022) A widespread family of phage-inducible chromosomal islands only steals bacteriophage tails to spread in nature 10.1016/j.chom.2022.12.001 36596306

[88] Ibarra-Chávez R, Reboud J, Penadés JR, Cooper JM (2023) Phage‐inducible chromosomal islands as a diagnostic platform to capture and detect bacterial pathogens. Adv Sci 10:2301643. 10.1002/advs.202301643 10.1002/advs.202301643 PMC10460865 37358000

[89] Mageeney CM, Lau BY, Wagner JM et al (2020) New candidates for regulated gene integrity revealed through precise mapping of integrative genetic elements. Nucleic Acids Res 48:4052–4065. 10.1093/nar/gkaa156 32182341 10.1093/nar/gkaa156 PMC7192596

[90] Eppley JM, Biller SJ, Luo E et al (2022) Marine viral particles reveal an expansive repertoire of phage-parasitizing mobile elements. Proc Natl Acad Sci U S A 119:e2212722119. 10.1073/pnas.2212722119 36256808 10.1073/pnas.2212722119 PMC9618062

[91] de Sousa JAM, Fillol-Salom A, Penadés JR, Rocha EPC (2023) Identification and characterization of thousands of bacteriophage satellites across bacteria. Nucleic Acids Res 51:2759–2777. 10.1093/nar/gkad123 36869669 10.1093/nar/gkad123 PMC10085698

[92] Fillol-Salom A, Rostøl JT, Ojiogu AD et al (2022) Bacteriophages benefit from mobilizing pathogenicity islands encoding immune systems against competitors. Cell 185:3248-3262e.e20. 10.1016/j.cell.2022.07.014 35985290 10.1016/j.cell.2022.07.014

[93] Humphrey S, San Millán Á, Toll-Riera M et al (2021) Staphylococcal phages and pathogenicity islands drive plasmid evolution. Nat Commun 12:5845. 10.1038/s41467-021-26101-5 34615859 10.1038/s41467-021-26101-5 PMC8494744

[94] Barcia-Cruz R, Goudenège D, De Moura JA et al (2024) Phage-inducible chromosomal minimalist islands (PICMIs), a novel family of small marine satellites of virulent phages. Nat Commun 15:664. 10.1038/s41467-024-44965-1 38253718 10.1038/s41467-024-44965-1 PMC10803314

[95] He L, Patkowski JB, Miguel-Romero L et al (2025) Chimeric infective particles expand species boundaries in phage inducible chromosomal island mobilization 10.1016/j.cell.2025.08.019 40930093

[96] Tao J, Wang S, Liao T, Luo H (2021) Evolutionary origin and ecological implication of a unique *nif* island in free-living *Bradyrhizobium* lineages. ISME J 15:3195–3206. 10.1038/s41396-021-01002-z 33990706 10.1038/s41396-021-01002-z PMC8528876

[97] Zhang N, Jin C-Z, Zhuo Y, Jin F-J, Jin L et al (2023) Genetic diversity into a novel free-living species of *Bradyrhizobium* from contaminated freshwater sediment. Front Microbiol 14:1295854. 10.3389/fmicb.2023.1295854 38075887 10.3389/fmicb.2023.1295854 PMC10708946

[98] Jin C-Z, Wu X-W, Zhuo Y, Jin F-J, Jin L et al (2022) Genomic insights into a free-living, nitrogen-fixing but non nodulating novel species of *Bradyrhizobium sediminis* from freshwater sediment: three isolates with the smallest genome within the genus *Bradyrhizobium*. Syst Appl Microbiol 45:126353. 10.1016/j.syapm.2022.126353 36030678 10.1016/j.syapm.2022.126353

[99] Tao J, Wang S, Liao T, Luo H (2021) Horizontal gene transfer of a unique *nif* island drives convergent evolution of free-living N_2_ -fixing *Bradyrhizobium*

[100] Melnyk RA, Hossain SS, Haney CH (2019) Convergent gain and loss of genomic islands drive lifestyle changes in plant-associated *Pseudomonas*. ISME J 13:1575–1588. 10.1038/s41396-019-0372-5 30787396 10.1038/s41396-019-0372-5 PMC6776051

[101] Arashida H, Odake H, Sugawara M et al (2022) Evolution of rhizobial symbiosis islands through insertion sequence-mediated deletion and duplication. ISME J 16:112–121. 10.1038/s41396-021-01035-4 34272493 10.1038/s41396-021-01035-4 PMC8692435

[102] Tang Y, Zhan P, Wu Y et al (2025) Landscape of mobile genetic elements and their functional cargo across the gastrointestinal tract microbiomes in ruminants. Microbiome 13:162. 10.1186/s40168-025-02139-1 40652256 10.1186/s40168-025-02139-1 PMC12255022

[103] Huang R, Ding J, Guo Y et al (2022) Habitat determines the relationships among bacteria, resistance genes and mobile genetic elements in the soil–plant system. Eur J Soil Sci 73:e13132. 10.1111/ejss.13132

[104] Guo J, Aroney S, Dominguez-Huerta G et al (2025) Mobile genetic elements that shape microbial diversity and functions in thawing permafrost soils 10.1038/s41564-026-02391-7 PMC13323075 42373820

[105] Cury J, Oliveira PH, De La Cruz F, Rocha EPC (2018) Host range and genetic plasticity explain the coexistence of integrative and extrachromosomal mobile genetic elements. Mol Biol Evol 35:2230–2239. 10.1093/molbev/msy123 29905872 10.1093/molbev/msy123 PMC6107060

[106] Ku Y-S, Wang Z, Duan S, Lam H-M (2021) Rhizospheric communication through mobile genetic element transfers for the regulation of microbe–plant interactions. Biology (Basel) 10:477. 10.3390/biology10060477 34071379 10.3390/biology10060477 PMC8227670

[107] Che Y, Yang Y, Xu X et al (2021) Conjugative plasmids interact with insertion sequences to shape the horizontal transfer of antimicrobial resistance genes. Proc Natl Acad Sci U S A 118:e2008731118. 10.1073/pnas.2008731118 33526659 10.1073/pnas.2008731118 PMC8017928

[108] Horne T, Orr VT, Hall JP (2023) How do interactions between mobile genetic elements affect horizontal gene transfer? Curr Opin Microbiol 73:102282. 10.1016/j.mib.2023.102282 36863168 10.1016/j.mib.2023.102282

[109] Deng Z, Zhao Y, Ren Z, Zhao W et al (2025) Ecological distribution, dissemination potential, and health risks of antibiotic resistance genes and mobile genetic elements in soils across diverse land-use types in China. Environ Res 285:122459. 10.1016/j.envres.2025.122459 40744195 10.1016/j.envres.2025.122459

[110] Bograd A, Oppenheimer-Shaanan Y, Levy A (2025) Plasmids, prophages, and defense systems are depleted from plant microbiota genomes. Genome Biol 26:163. 10.1186/s13059-025-03641-3 40500753 10.1186/s13059-025-03641-3 PMC12153167

[111] Ghaly TM, Gillings MR (2022) New perspectives on mobile genetic elements: a paradigm shift for managing the antibiotic resistance crisis. Philos Trans R Soc B Biol Sci 377:20200462. 10.1098/rstb.2020.0462 10.1098/rstb.2020.0462 PMC8628067 34839710

